# Testing the Challenge Hypothesis in Stumptail Macaque Males: The Role of Testosterone and Glucocorticoid Metabolites in Aggressive and Mating Behavior

**DOI:** 10.3390/biology12060813

**Published:** 2023-06-03

**Authors:** Ana Lilia Cerda-Molina, Javier I. Borráz-León, Gilberto Matamoros-Trejo, Claudio de la O, Gema R. Estudillo-Mendoza, Lilian Mayagoitia-Novales, Dario Maestripieri

**Affiliations:** 1 Departamento de Etología, Instituto Nacional de Psiquiatría “Ramón de la Fuente Muñiz”, Ciudad de México 14370, Mexico; alcm@imp.edu.mx (A.L.C.-M.); gem@imp.edu.mx (G.R.E.-M.); mayagn@imp.edu.mx (L.M.-N.); 2 Institute for Mind and Biology, The University of Chicago, Chicago, IL 60637, USA; dario@uchicago.edu; 3 Departamento de Neurofisiología Molecular, Instituto Nacional de Psiquiatría “Ramón de la Fuente Muñiz”, Ciudad de México 14370, Mexico; gmtrejo@imp.edu.mx; 4 FES Zaragoza C-III, Universidad Nacional Autónoma de México, Santa Cruz Tlaxcala 90640, Mexico; claudio.delao@zaragoza.unam.mx; 5 Escuela de Psicología, Universidad Latina, Ciudad de México 4330, Mexico; 6 Department of Comparative Human Development, The University of Chicago, Chicago, IL 60637, USA

**Keywords:** fecal glucocorticoid metabolites, fecal testosterone metabolites, challenge hypothesis, aggression, copulation, *Macaca arctoides*

## Abstract

**Simple Summary:**

In most primates, the “challenge hypothesis” predicts increases in male–male aggressive behavior and testosterone during reproductive challenges, i.e., mating competition, or during periods of social instability, especially in seasonal breeders. However, few studies have investigated the role of stress hormones, such as glucocorticoids, and social rank in non-seasonal primates. In this study, we tested some predictors of the “challenge hypothesis” during a period of social stability in a non-seasonal primate. We collected data on aggressive behavior (male-to-male and male-to-female), copulation, and fecal samples (n = 700) to quantify testosterone and glucocorticoid metabolites (fTm and fGCm, respectively) in seven adults of stumptail macaques (*Macaca arctoides*) living in semi-captivity. We found that fTm and fGCm increased during times of copulation, although neither of the two hormones were associated with aggression between males. Besides, fGCm levels were positively associated with male-to-female aggression, indicating a possible social stress challenge. Both fTm and fGCm levels were higher in higher- and middle-ranking males. Our findings partially support the “challenge hypothesis” in stumptail males and highlight the importance of glucocorticoid secretion during challenging situations; our results also indicate that during periods of social stability, testosterone is not associated with aggression, at least in a non-seasonal primate.

**Abstract:**

The “challenge hypothesis” predicts higher male–male aggressive behavior along with increases in testosterone levels during times of reproductive challenges and social instability. In addition, in some primate species, higher glucocorticoid levels can be observed as well, but this is usually modulated by dominance rank. We studied rank-related aggressive behavior, mating activity, and fecal testosterone and glucocorticoid metabolites (fTm and fGCm) in male stumptail macaques (*Macaca arctoides*) in order to test some predictions of the “challenge hypothesis”. Over a 20-month period, we collected data on aggressive behavior and copulation, as well as fecal samples (n = 700) to quantify fTm and fGCm in seven adult stumptail males living in captivity. During periods of mating activity, male-to-male aggression increased in higher- and middle-ranking males. Neither fTm nor fGCm levels predicted male-to-male aggression. fGCm levels (but not fTm) were positively associated with male-to-female aggression; however, this association was pronounced during periods of mating activity. fGCm levels differed according to social rank, with middle-ranking males having the highest levels. Both hormones were higher during periods of mating activity, but only in higher- and middle-ranking males. Taken together, our findings partially support the challenge hypothesis in a non-seasonal primate and shed some light on the unique social and mating system of the stumptail macaque.

## 1. Introduction

Aggression includes a repertoire of behaviors widely used throughout the animal kingdom as an offensive or defensive strategy for the protection of individuals and groups as well as to maintain social order [1]. Aggressive behavior is common among group-living individuals, typically—but not unavoidably—occurring when incompatible goals among group members clash [2] and ranges from harmless (e.g., threats, intimidations) to potentially harmful physical displays [3]. 

From a functional perspective, aggressive behavior can be viewed as a competitive strategy favored when the benefits of escalating a fight exceed its potential costs, e.g., [2,4]. Regarding proximate causation, previous research has extensively documented the role of environmental, e.g., [5,6]; neural, e.g., [7,8,9]; and hormonal mechanisms in aggressive behavior, e.g., [8,10,11].

In non-human primates, aggression takes place in several contexts such as over territories, social status, food, or mating opportunities [2]. In particular, competition for reproductive opportunities can create a challenging situation in which males confront each other—sometimes in a highly aggressive way—to gain access to receptive females [12,13]. In this fitness-relevant context, it is well known that male hormones such as testosterone (T) regulate behavioral aspects of mating efforts through enhancing the motivation for male–male competition and male–female courtship [14]. 

Male T is involved in the development of reproductive organs, secondary sexual characteristics, the regulation of male sexual and courtship behavior, and, in some species, the motivation for social status competition [15,16,17]. In some species of non-human primates, adult males with the highest levels of T are more likely to monopolize fertile females [8,18,19,20,21]. Much of the existing evidence linking T and aggression in males has been explained under the framework of the “challenge hypothesis”, originally proposed and documented in birds and later adapted to human and non-human primates [12,22,23]. 

The challenge hypothesis has many predictions: (1) during mating season, T will rise as a response to sexual stimuli of receptive females; (2) the increase in T motivates males to compete for access to receptive females and provides metabolic energy for courtship and fighting; (3) at the end of the breeding season, T decreases [10,13,14] and males become unresponsive to females sexual solicitations, e.g., when these are experimentally induced with estrogen treatment [24]; (4) T remains at low levels while males are involved in paternal care (e.g., in monogamous birds and in humans) and in conjunction with a rise in prolactin and oxytocin [25]. 

In support of the “challenge hypothesis”, many studies of seasonal primate species have documented increases in levels of fecal testosterone metabolites (fTm) during the breeding season, which were positively associated with the frequency of copulation and with the intensity of male aggressive displays [18,19,22,26]. In seasonal species such as rhesus macaques (*Macaca mulatta*), high levels of T, frequent fighting and injuries, and energy depletion for courtship during the mating season are also associated with high male mortality [27]. In primates, the relationship between competitive aggression and T is often modulated via the dominance hierarchy. For instance, in baboons and rhesus macaques, the magnitude of the elevation in T and aggression may be greater when dominance relationships are challenged and the hierarchy is unstable than when the hierarchy is stable and unchallenged [11,15,28,29,30,31,32,33]. Male courtship and fighting are also energetically expensive and socially stressful; thus, the mating season is also associated with increased secretion of glucocorticoids (GCs), particularly cortisol [1]. 

There is variation in the pattern and intensity of the endocrine responses during intrasexual competition in males depending on many factors such as the species, the type of dominance hierarchy (despotic vs. egalitarian, stable vs. unstable), the pattern of reproduction (seasonal vs. non-seasonal), the mating system, the number of males in a group, and the number of females that may come into estrus simultaneously on a given day [1]. For example, in the ring-tailed lemur (*Lemur catta*), a seasonal breeding prosimian, fecal T metabolites (fTms) and aggression rates in males are positively correlated only during the mating season [13]. In the seasonally breeding Assamese macaque (*Macaca assamensis*), male fecal epiandrosterone levels—a T metabolite—increase during the mating period along with intra-male aggression. Furthermore, since lower-ranking males usually receive more aggression, they have higher fecal GC metabolite (fGCm) levels than dominant males [19,34]. Among *Macaca fascicularis*, a primate with a moderate degree of reproductive seasonality, Girard-Buttoz et al. [18] found that fTms, but not fGCms, begin to increase one or two months before the onset of the mating period, in association with elevated rates of inter-male aggression. Regarding non-seasonal breeders, Gesquiere et al. [35] found that dominant males in a wild savannah baboon population (*Papio cynocephalus*) have higher fTm but lower fGCm levels than lower-ranking males; however, the exception was the alpha male who maintained the highest levels of both hormones, suggesting a higher energetic cost of being at the top of the hierarchy. In chimpanzees (*Pan troglodytes schweinfurthii*), the excretion of both fGCms and fTms is correlated positively with higher rates of male aggression, and the highest concentrations are associated with those times when parous females display maximally tumescent sexual swellings [22,23]. 

In summary, the common pattern in male primates is to increase T secretion during times of intense mating competition and inter-male aggression; however, GCs may increase only in subordinate individuals, not increase at all, or increase only in the alpha male. Because maintaining high T levels for long periods of time can increase mortality through increasing the metabolic rate and suppressing immune function, e.g., [27], non-seasonal species should show only transient rises in T when challenging situations make this hormone necessary or useful. 

Our animal model, the stumptail macaque (*Macaca arctoides*), lives in multimale/multifemale social groups with linear dominance hierarchies in both free-ranging [36] and captive populations [37,38,39]. Previous studies have suggested that, despite relatively low levels of damaging aggression, the stumptail macaque is a highly despotic species, in which the alpha male or the two highest-ranking males can greatly intimidate other males and monopolize receptive females [36,40,41,42,43]. Furthermore, most studies have confirmed that stumptail macaques mate year-round and do not show reproductive seasonality [37,44,45,46]. Female stumptail macaques do not show exaggerated sexual swellings, unlike other macaque species, baboons, and chimpanzees [47,48]. Stumptail males, however, may assess the receptivity of females through approaching and sniffing the genital region, together with intense—and sometimes aggressive—vaginal inspections [49,50]. In a recent study, Cerda-Molina et al. [46] investigated male aggression against females as a potential sexual coercion strategy. It is possible that, in the presence of oestrus females, high-ranking stumptail males may show transient increases in T (to enhance mating motivation and aggressive ‘courtship’). It is also possible that middle- or lower-ranking males may show increases in GC levels due to the potential for male–male aggression and displacement. These issues have not been explored in previous studies. Moreover, addressing the relationship between T, GC, and aggression in a non-seasonal breeder such as the stumptail macaque can shed light on the generality of the challenge hypothesis and its applicability to non-seasonal species. 

The main goal of the present study was to test some predictions of the “challenge hypothesis” in stumptail macaques, with data concerning both aggressive behavior and fecal hormone metabolites. Specifically, we studied seven focal males in a captive group to explore whether (1) focal males’ aggression towards other males changes according to the male’s fTm and fGCs levels, rank category (higher, middle, or lower ranking), mating context (copulation vs. no copulation), or the target of aggression (i.e., juvenile/subadult or adult males); (2) focal males’ aggression towards females changes according to males’ fTm and fGCm levels, rank category (higher, middle, or lower ranking), mating context (copulation vs. no copulation), or with the target of aggression (i.e., juvenile/subadult or adult females). Additionally, we studied whether fTm and fGCm levels vary in relation to age, rank, and mating activity. We predicted that both male-to-male aggression and male-to-female aggression would be positively associated with higher male fTm and fGCm, with this association being stronger during mating activity (i.e., copulation context) and probably more prominent in higher ranking males. We also predicted that higher-ranking males would have the highest levels of fTms, while the lowest-ranking ones would have the highest levels of fGCms. Regarding the target of aggression, we hypothesized that males would direct more aggression towards juvenile/subadult males, as the latter may pose more of a threat in terms of rank and mating competition, and males would direct more aggression towards juvenile/subadult females, as the latter might be more sexually attractive due to their age and novelty.

## 2. Materials and Methods

### 2.1. Subjects and Housing Conditions

The focal subjects of this study were seven adult males of stumptail macaques, aged between 10 and 23 years (see Table 1), that were part of a captive group with 28 individuals (7 adult males, 3 juvenile males, 1 infant male, 14 adult females, 2 subadult females, and 1 infant female) at the time of the study. The animals were maintained in three large trapezoid outdoor facilities which were connected to each other (6.2 m length × 1.7 m minor side × 6 m major side × 6 m height for each one) and maintained under naturalistic environmental conditions (i.e., ambient temperature, humidity, and photoperiod) at the Ethology Department of the Instituto Nacional de Psiquiatría “Ramón de la Fuente Muñiz” in Mexico City, Mexico. The space was provided with six 1.5 m-deep × 6 m-long × 1.8 m-high platforms, some chains, a swing, and kindergarten playground objects such as a slide and a running wheel. The outdoor facilities were cleaned daily from 07:00 to 09:00 h; afterward, the animals were fed an early meal of fresh fruits and vegetables, and a midday meal of monkey processed food (Lab Diet 5038, PMI Feeds, Inc., St Louis, MO, USA). Clean tap water was available ad libitum.

### 2.2. Fecal Sample Collection 

We analyzed hormonal concentrations of only seven adult males whose fecal samples were reliably identified and collected on a continuous basis immediately after defecation. Fecal samples were collected between 10:00 and 12:00 h every 2–3 days (from Tuesday to Friday) from May 2010 to December 2011. We could not obtain daily fecal samples, since we collected only those feces that were observed and not contaminated with urine. We collected a total of 700 fecal samples (between 57 and 129 per individual, Table 1). The fecal samples were collected in conic polypropylene tubes (15 mL) and were immediately dried in a thermo savant vacuum concentrator at 60 °C (around 8–10 h), pulverized, and frozen (−70 °C) until assayed. 

### 2.3. Hormone Extraction and Quantification

We applied a methanol extraction following the method described by Pineda-Galindo et al. [39] for *Macaca arctoides.* Briefly, we mixed 0.5 g of dried, pulverized, and sieved feces with 4 mL of 100% methanol in conic polypropylene tubes (15 mL); we shook the tubes in a vortex for 3 min. After that, we placed the tubes for 24 h in a vertical shaker. The next day, we centrifuged the samples (1500× *g* for 30 min at 4 °C) and recovered the supernatants in 10 mL glass tubes. We added 1 mL of methanol to the remaining fecal pellets, shook the samples in a vortex, and centrifuged them again. We mixed both supernatants, evaporated to dryness (in a water bath at 60 °C), and reconstituted in 1 mL of phosphate buffer pH 7 (0.01 M, diluted with absolute ethanol 2:1 and Tween 20, 0.02% solution). To measure fTms, we diluted the samples 1:40 in the above-mentioned phosphate buffer pH 7; samples for fGCm measurement did not need to be diluted. We used commercially supplied radioimmunoassay kits (^125^ I RIA Kit Coat-A-Count, DPC, Los Angeles, CA, USA). For fTms, the analytical sensibility was as follows: 4 ng/dl; standard curve adjustment was R^2^ = 0.99; inter-assay precision in higher and lower concentrations was 7.3% (*n* = 10) and 11% (*n* = 10), respectively; and intra-assay precision in higher and lower concentrations was 6.6% (*n* = 10) and 10.95% (*n* = 10), respectively. For fGCms, the analytical sensibility was as follows: 3.62 ng/mL, standard curve adjustment was R^2^ = 0.98; inter-assay precision in higher and lower concentrations was 7.62% (N = 10) and 11.45% (*n* = 10), respectively; and intra-assay precision in higher and lower concentrations was 7.3% (*n* = 10) and 10% (*n* = 10), respectively. We expressed concentrations as ng/g of dry fecal matter. 

### 2.4. Behavioral Analyses

A research assistant conducted two daily sessions of scanning sampling for all subjects (from 12:00 to 13:00 h and 15:00 to 16:00). Each subject from the colony (i.e., the 28 macaques) was observed and their behavior recorded for around 10–15 s in a randomly designated; since every scanning lasted around 7–8 min, we completed six scans, every 10 min, until completing the hour. We also simultaneously collected ad libitum data on aggression and copulation frequency (only male-to-female) [51]. Observations were made from May 2010 to December 2011 (approximately 760 total focal hours). Although we conducted daily behavioral observations, we built our database using the frequency of aggression/h of the days in which fecal samples were successfully collected in order to associate hormone levels with behavior during the same time periods (we considered the 24–28 h of lag of excretion of hormones previously reported by Pineda-Galindo et al. [39]). We created a dominance sociomatrix that combined the frequency of aggressions and submissions exchanged between each pair of individuals. The aggressive behaviors recorded were slapping, staring, open mouth, grabbing, pulling, pushing, mock biting, biting, lunging, pretend slapping, pulling fur, and chasing; the submissive behaviors were teeth chattering, avoiding, crouching, fleeing, silent bared teeth, and hip presentation when preceded by aggression [40,52,53]. Then, we calculated the Normalized David’s scores (NormDS) every six months [54] (Table 1) and the corrected Landau Index h’ of linearity accounting for the number of unknown relationships [55,56]. The higher the NormDS value, the higher the social rank, while Landau Index h’ informs on the predictability and steepness of dominance relationships among individuals. NormDS and linearity were calculated using the ‘compete’ libraries for R package 0.1 designed by Curley [57] and computed in R 3.5.1 [58]. We obtained behavioral data during a stable social situation in which no dominance challenge occurred, i.e., the alpha and beta males remained the same during the study period (Table 1). In our analyses, we classified the two top-ranked individuals (the alpha and beta males) with the highest NormDS values as high-ranking according to Richter et al. [36]; we classified the next three males as middle-ranking and the two males with the lowest NormDS values as lower-ranking (Table 1). We constructed a database that matched days with fecal hormone concentration and aggression frequency (*n* = 700 data points). 

### 2.5. Data Analyses

We first explored data on the frequency of aggression (shown by the 7 focal males) directed to other males and females through performing nonparametric Friedman tests. To test the predictions of the “challenge hypothesis”, we built two Generalized Estimated Equation (GEE) models, one for male-to-male aggression (focal male aggression directed to other males in the group as dependent variable), and a second for the male-to-female aggression (frequency of focal male aggression directed to females as dependent variable). GEE is suitable for dealing with autocorrelated data, i.e., repeated measures on the same subjects with the option to select the appropriate distribution link function to analyze the dependent variables [59,60]. As predictors, we included male rank (high, middle, and low-ranking), mating context (as binomial variable copulation / no copulation), the target of aggression (via categorizing targets as subadults / juveniles or adults), and fTm and fGCm levels (log-transformed). We analyzed the interactions: rank × target of aggression, mating context × rank, mating context × fTm levels, and mating context × fGC levels. We used the Tweedie probability distribution with the log link function since the behavioral frequencies did not comply with normality and our data were a mixture of zeros (i.e., absence of any aggression during the sampling hours) and positive values [61]. In all models, we included monkey ID as the subject variable and selected the auto-regressive correlation structure which is suitable for regularly repeated measurements on the same subject [60]. We performed two additional GEE models to explore the effect of mating context and rank on fecal hormone metabolites. Where appropriate, we applied Bonferroni post hoc tests; all results are described as means (M) and 95% confidence intervals (CI). All the analyses were performed on IBM SPSS version 22; significance was set at *p* ≤ 0.05.

## 3. Results

### 3.1. Aggression

Focal males directed significantly more aggression toward females (M = 5.62, 95%CI [4.62–6.62]) than toward other males (M = 3.05, 95%CI [2.33–3.42]) (Friedman χ^2^ = 33.247, df = 1, 293, *p* < 0.001). Total aggression varied according to the social rank of males (GEE Wald χ^2^ = 41.937, d.f. = 2, 699, *p* < 0.001). As expected, higher-ranking males displayed more aggressive behaviors than middle- (M = 4.14, 95%CI [3.04–5.26] vs. 1.98, 95%CI [1.60–2.36], *p* = 0.003) and lower-ranking males (M = 4.14, 95%CI [3.04–5.26] vs. 0.97, 95%CI [0.67–1.28], *p* < 0.001). Middle-ranking males displayed more aggression than lower-ranking males (M = 1.98, 95%CI [1.60–2.36] vs. 0.97, 95%CI [0.67–1.28], *p* = 0.036). 

### 3.2. Male-to-Male Aggression

**Figure 1:**
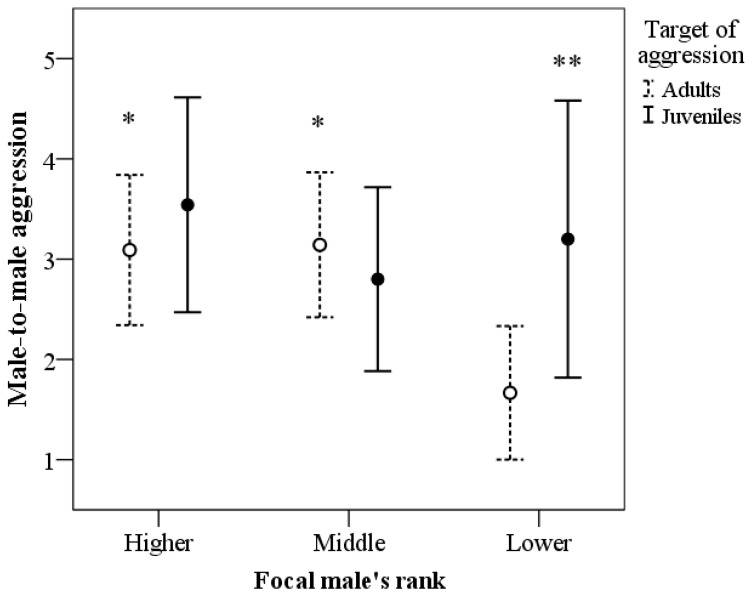
Frequency of aggression of the seven focal males and the target of aggression (adult [N = 90] or juvenile males [N = 61]) and according to the focal male’s rank. Data represent mean and 95% CI. ** *p* < 0.01 juveniles vs. adults; * *p* < 0.05 vs. Lower-ranking males.

**Figure 2:**
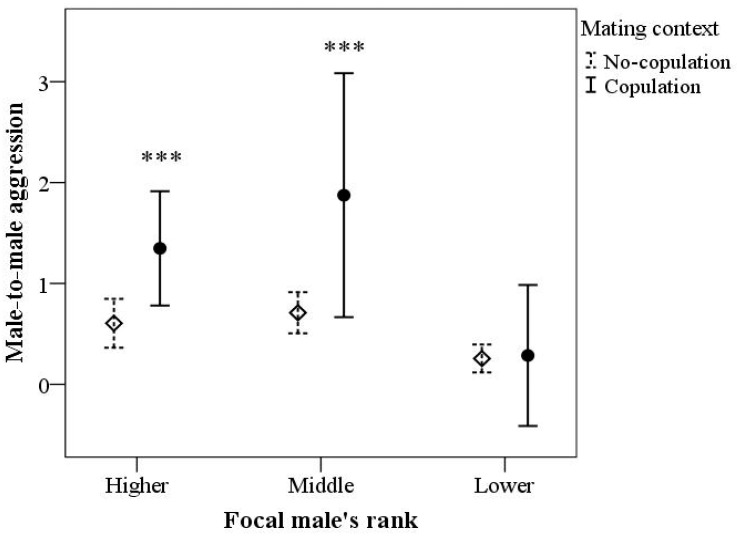
Frequency of aggression of the seven focal males according to social rank and mating activity (copulation [N = 40] vs. no copulation with females [N = 111]). Data represent mean and 95% CI. *** *p* < 0.001 vs. lower-ranking males during the context of mating activity.

Table 2 shows the results of the GEE model for male-to-male aggression. There was a significant effect of the target of aggression (*p* < 0.001), the interaction target of aggression × male’s rank (*p* < 0.001), and the interaction mating context × male’s rank (*p* < 0.001). The other main and interaction effects were not statistically significant. Figure 1 indicates that higher- and middle-ranking males displayed more aggression towards adult males, compared to lower-ranking males (*p* = 0.013 and *p* = 0.035, respectively); instead, lower-ranking males displayed a higher frequency of aggression toward juvenile males compared to adults (*p* = 0.004). Figure 2 shows that higher- and middle-ranking males displayed significantly more aggression than lower-ranking males, but only during the copulation context (*p* < 0.001 in both cases). There were no significant differences (*p* = 1.00) in aggression frequency between higher and middle-ranking males in the mating context (copulation vs. no copulation). 

### 3.3. Male-to-Female Aggression

**Figure 3:**
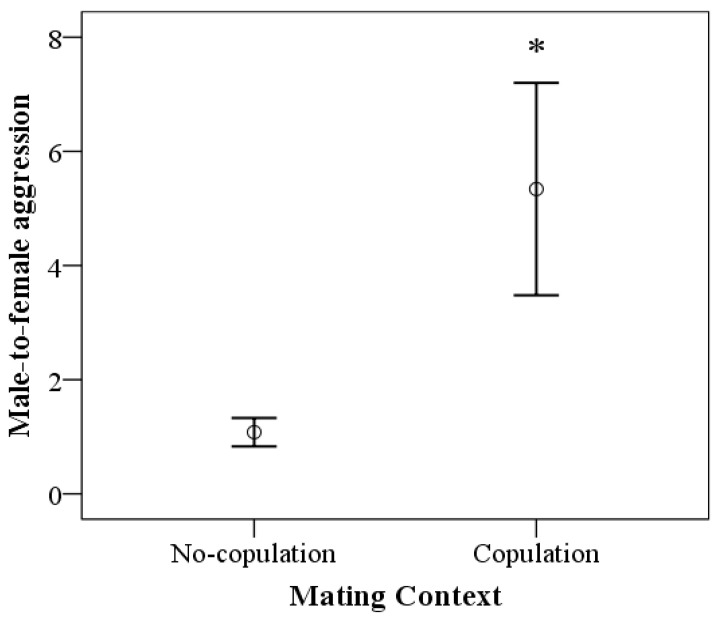
Frequency of aggression directed to females from the seven focal males according to mating activity (presence [N = 51] or absence of copulation with females [N = 151]). Data represent mean and 95% CI. * *p* = 0.05 vs. absence of mating activity.

**Figure 4:**
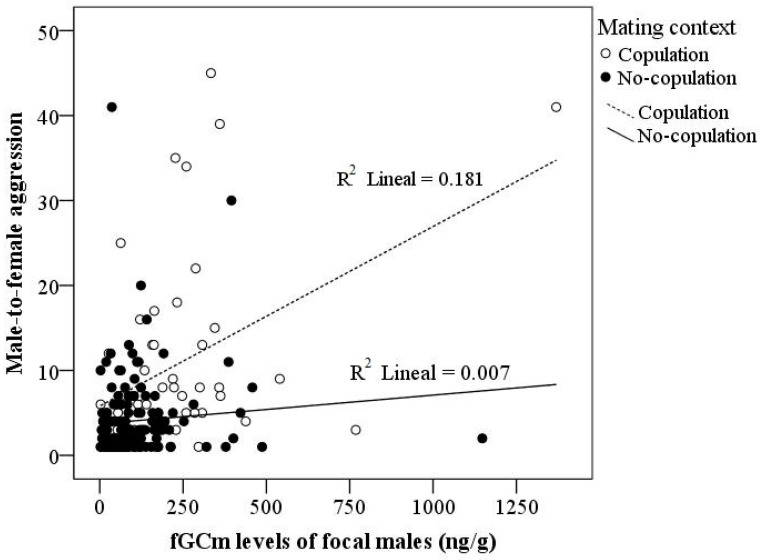
Relationship between males’ aggression directed to females and fecal glucocorticoid metabolite levels (fGCm) of the seven focal males according to mating activity (copulation [N = 99] vs. no copulation [N = 564].

The left side of Table 2 shows the results of the GEE model for male aggression directed toward females. We found a marginally significant effect of the mating context (*p* = 0.05), but a significant main effect of the target of aggression (*p* = 0.003), the focal male’s fGCm levels (*p* < 0.001), and the interaction mating context × fGCm levels (*p* = 0.003). The other variables and the interactions between them did not yield statistically significant results. Figure 3 shows that the frequency of aggression directed toward females was higher during the copulation context (*p* = 0.05). Figure 4 shows a positive relationship between the focal male’s fGCm levels and the frequency of aggression toward females; the association was positive only during the copulation context. 

### 3.4. Predictors of fTm and fGCm Levels

**Figure 5:**
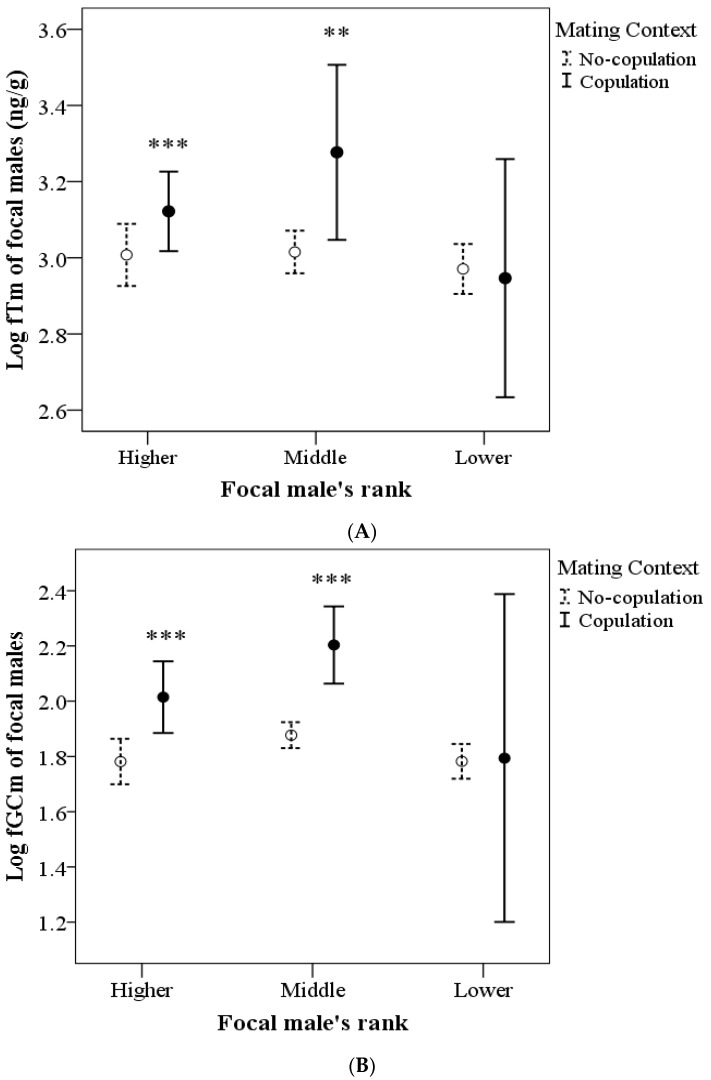
Comparison between (**A**) fecal testosterone (fTm) and (**B**) glucocorticoid metabolite (fGCm) levels of the seven focal males according to social rank and depending on mating activity (fTm: copulation [N = 100], no copulation [N = 600]; fGCm: copulation [N = 99], no copulation [N = 564]). Data represent mean and 95% CI. *** *p* < 0.001 vs. no-copulation, ** *p* < 0.01 vs. no-copulation.

Table 3 shows the results of GEE models for fecal hormone metabolites. Male fTm levels did not vary with rank or with age of focal males (*p* = 0.211 and *p* = 0.343, respectively), but varied according to mating context and with the interaction mating context × focal male rank (*p* = 0.004 and *p* = 0.048, respectively). Figure 5A shows that higher- and middle-ranking males had higher levels of fTms in the copulation context compared to other contexts (*p* < 0.001 in both cases), whereas lower-ranking males did not differ according to mating context (*p* = 1.0). 

The levels of fGCms did not vary with age (*p* = 0.738) but varied according to the focal male’s social rank (*p* < 0.001), the mating context (*p* < 0.001), and the interaction between mating context × focal male’s rank (*p* = 0.01). Figure 5B indicates that fGCm levels were also significantly higher in the copulation context in higher- and middle-ranking males (*p* < 0.001). Lower-ranking males did not differ between copulation vs. no-copulation contexts (*p* = 1.0). In general, middle-ranking males had the highest levels of fGCms compared with both higher- and lower-ranking males (*p* < 0.001); higher- and lower-ranking males did not differ in fGCm levels (*p* = 0.208).

## 4. Discussion

As expected, the higher and middle-ranking males increased their aggressive behavior toward other males during times of mating activity [37]. Contrary to our hypothesis, fTm levels were not associated with such an increase in male aggression; instead, fTm levels were high only during copulation times, supporting the role of T in the expression of courtship and sexual behavior in stumptail macaques [46].

Males were more aggressive toward females than toward other males. According to the sexual conflict model by Van Schaik et al. [62], males, particularly the higher-ranking ones, might be more aggressive toward females as a strategy to prevent them from mating with rival males, since this option may be less risky than attacking the rival males directly. Male aggression toward other males may also be energetically costly and stressful [2,23]. Although we did not find a direct association between fTms and aggression, we found that male-to-female aggression during times of copulation was associated with increased fGCm levels, which might support the notion that aggression and mating activity are challenging and energetically costly to males. 

It is well known that stressful or challenging situations trigger the activation of the HPA axis, which results in the release of GCs. One of the main functions of GCs is to increase the circulating levels of glucose to make available the energy needed to mount a behavioral response to the challenge [63]. It is possible that, in addition to the psychosocial stress associated with elevated intrasexual competition, mating also increases GCs, creating a metabolic stress response [23]. 

Our results concerning higher levels of male fGCms together with aggression during times of mating activity are consistent with the challenge hypothesis and are similar to those reported for other seasonal and non-seasonal primate species. For instance, in the seasonal white-faced capuchin (*Cebus capucinus*), it has been shown that male fTm and fGCm levels are higher in the presence of fertile females, regardless of male rank and age [64]. In the seasonally breeding Assamese macaque (*Macaca assamensis*), an increase in aggressive behavior occurs during the mating season concurrent with an increase in fGCm levels, even though the hormone levels were negatively correlated with social rank [34]. In the non-seasonal chimpanzee (*Pan troglodytes schweinfurthii*), both fGCm and fTm levels are higher during times of female maximally tumescent sexual swellings [22,23].

The finding that fTms arose with mating activity without being associated with male aggression might suggest an important role of T in sexual motivation to copulate. In the present study, general fluctuations of male fTm levels were not associated with rank or age, although variations in hormone levels according to the mating context were evident for higher- and middle-ranking males but not for lower-ranking ones. These findings might be due to the fact that we studied a group of adult macaques (i.e., ≥10 years old) and major fluctuations are reported for adolescent individuals; thus, it is possible that a relationship between fTms and aggression in the context of mating activity is strongest in younger males [11,65]. Furthermore, our study group was highly stable, with no changes in the hierarchy of the two top-ranking males; under these conditions, no significant associations between testosterone and aggression are expected [12].

In the mating context, stumptail males increased their aggression toward the females, presumably as an expression of a coercive sexual strategy (see also Cerda-Molina et al. [46] for stumptails and Reed at al. [66] for Sulawesi crested black macaques, *Macaca nigra*). Lower-ranking males, however, displayed more aggression toward juvenile than adult males, that is, toward individuals that are lower ranking than themselves. Thus, both sexual and social strategies (to maintain or enhance one’s rank in the hierarchy) were at play among the males and were expressed through aggressive behavior [37]. 

Although some previous studies reported rank-related changes in glucocorticoid levels in association with male–male mating competition [35,67,68], in our study, the middle-ranking males had the highest levels of fGCms. This result could be related to the dynamics of male social interactions to maintain social rank. For example, compared to higher- and lower-ranking males, the middle-ranking ones were the least stable in their ranks (as evaluated via the NormDS). We also found that higher- and middle-ranking males, but not lower-ranking males, showed increased levels of fGCm during mating activity, which might be explained by the fact that lower-ranking males rarely copulate with females, since copulations are usually monopolized by the most dominant individuals. The lowest-ranking males may have the chance to copulate with the lowest-ranking females, but only when the higher-ranking males are out of sight [47]. Furthermore, some previous studies have reported a less intense HPA-axis activation among low-ranking monkeys because they are habituated to the chronic stressor of being constantly attacked by higher-ranking animals [39,69]. The present study had the limitation of a small sample size of focal males, which might constrain our interpretation of the behavioral data and our ability to make generalizations concerning age classes or social ranks; this is especially true for the lowest-ranking males, who displayed the lowest aggression frequencies.

## 5. Conclusions

Taken together, the results of this study support some predictions of the challenge hypothesis among male stumptail macaques, suggesting that fluctuations in male fGCm levels are related to aggression displays during mating activity and that male fTm levels may function primarily in the context of male motivation to copulate with females. We also showed that these behavioral and hormonal changes vary in relation to the social rank of the individual. Our findings contribute to our understanding of the challenge hypothesis (and its applicability to both seasonal and nonseasonal primate species) as well as to our knowledge of the complex and unique social and mating system of the stumptail macaque.

## Figures and Tables

**Table 1:** Sociodemographic characteristics of stumptail males (age and social rank) at the beginning of sampling and the number of fecal samples collected from the focal males. Normalized David scores (NormDS) are presented for each semester.

Males	Age in Years (Class)	NormDS 2010 *h*’ = 0.75 ^a^	NormDS 2011-1 *h*’ = 0.83 ^a^	NormDS 2011-2 *h*’ = 0.63 ^b^	No. of Fecal Samples
DF ^1^	10 (adult)	9.62	9.62	8.61	104
AL ^1^	20 (adult)	8.44	8.60	7.85	117
JI ^2^	16 (adult)	6.58	7.21	5.59	110
ES ^2^	20 (adult)	5.60	6.42	5.57	129
PO ^2^	22 (adult)	5.03	4.44	5.10	57
RP	3 (juvenile)	4.91	3.37	4.34	
PL	1 (infant)	3.88	3.79	4.04	
FO	3 (juvenile)	3.72	3.53	4.18	
GA ^3^	16 (adult)	3.40	3.27	3.95	87
RE	3 (juvenile)	3.06	3.48	3.43	
DW ^3^	23 (adult)	0.75	1.29	2.33	96

^1^ Higher-ranking; ^2^ Middle-ranking; ^3^ Lower-ranking. ^a^ *p* < 0.001; ^b^ *p* = 0.07.

**Table 2:** Results of the GEE models for male aggression of the seven focal stumptail males.

	Focal Male-to-Male Aggression			Focal Male-to-Female Aggression		
Predictor	Wald χ^2^	d.f.	*p*	Wald χ^2^	d.f.	*p*
Interception	0.464	1	0.496	0.341	1	0.560
Mating context	0.854	1	0.355	3.826	1	**0.050**
Target of aggression	68.257	1	**<0.001**	8.724	1	**0.003**
Focal male’s fTm	2.597	1	0.107	2.832	1	0.092
Focal male’s fGCm	0.049	1	0.825	33.349	1	**<0.001**
Mating × rank	14.113	2	**0.001**	1.851	2	0.829
Mating × fTm	1.239	1	0.266	0.000	1	0.985
Mating × fGCm	0.041	1	0.839	8.716	1	**0.003**
Target of aggression × rank	31.398	2	**<0.001**	0.376	2	0.829

Note: fTm = fecal testosterone metabolites; fGCm = fecal glucocorticoid metabolites.

**Table 3:** Results of the GEE models for fecal hormone metabolites of the seven focal stumptail males.

	**Fecal Testosterone Metabolites**			**Fecal Glucocorticoid Metabolites**		
**Variable**	**Wald χ^2^**	**d.f.**	***p***	**Wald χ2**	**d.f.**	***p***
Interception	488.608	1	0.000	104.595	1	0.000
Age	0.901	1	0.343	0.151	1	0.697
Rank	3.112	2	0.211	48.422	2	**<0.001**
Mating Activity	8.248	1	**0.004**	31.594	1	**<0.001**
Mating × rank	6.072	2	**0.042**	9.305	2	**0.010**

## Data Availability

All data generated or analyzed during this study are included in this published article.
